# Effect of Red Arch-Support Insoles on Subjective Comfort and Movement Biomechanics in Various Landing Heights

**DOI:** 10.3390/ijerph17072476

**Published:** 2020-04-05

**Authors:** Yi Wang, Wing-Kai Lam, Cheuk-Hei Cheung, Aaron Kam-Lun Leung

**Affiliations:** 1 State Key Lab of Space Medicine Fundamentals and Application, China Astronaut Research and Training Centre, Beijing 100094, China; wyi@bsu.edu.cn; 2 China Institute of Sports and Health Science, Beijing Sport University, Beijing 100084, China; 3 Department of Kinesiology, Shenyang Sports Institute, Shenyang 110102, China; 4 Li Ning Sports Science Research Center, Beijing 101111, China; cheungcheukhei2002@gmail.com; 5 Department of Biomedical Engineering, The Hong Kong Polytechnic University, Hong Kong, China; aaron.leung@polyu.edu.hk

**Keywords:** basketball, foot insert, orthosis, injury prevention, footwear

## Abstract

Red is perceived as a “winning color”, which may influence actual and perceived performances in sports, but little effort has been done to assess the added value on colored foot insoles in basketball movements. This study examined if colored foot insole would influence perceived comfort and lower extremity biomechanics during drop landing. Nineteen male basketball players performed drop landing trials with different insoles (red arch-support, white arch-support, and white-flat) and landing heights (0.45 and 0.61 m). Two-way (Insole x Height) ANOVAs with repeated measures were performed on each of the knee and ankle angles and moments variables. Wearing red arch-support insoles induced better perception of forefoot and rearfoot cushioning and overall comfort but smaller plantarflexion moment than the white-flat insoles (*p* < 0.05). Increased landing height was related to higher ground reaction loading, sagittal flexion angles, range of motion, and joint moments but smaller ankle eversion (*p* < 0.05). Findings indicate that foot insoles might have influenced comfort perception and joint kinetics, but not joint kinematics. The use of red color in foot insoles could potentially maximize the effectiveness of foot insoles in a way that alters comfort perception and motor control during landing, with implications for risk of injury.

## 1. Introduction

The belief that the color red enhances winning percentages and better human performance has driven the training and application over the years (e.g., penalty kick training for soccer goalkeepers and penalty takers, [1,2,3]). Red color can enhance people’s aggressiveness and increase testosterone levels that improve the perception of dominance as well as physical tasks and sport performances [1,4,5]. Athletes wearing red increase the chance of winning in other colors [1,6], as indicated by outperformed performances in penalty kick accuracy and successful goalkeeper’s safe [2,3]. The previous studies reported that viewing red has been shown to increase handgrip force and velocity [7] and that participants wearing red jerseys had significantly higher heart rates and higher pre-contest leg strength than control colors [8]. However, previous color studies have focused predominantly on apparel effects (e.g., jersey) and did not report joint biomechanics that is related to performance and injury. If teams do not use a red uniform, the use of red color in sport accessories (e.g., shoes, insoles) may also influence their preference, movement biomechanics and performance. The previous study had participants performing maximum vertical jumps in different shoe colors, showing that wearing red shoes would lead to better jump take-off performance [9]. However, this study did not report the data for landing phase, it is questionable if the use of red color in footwear/insoles would also influence landing mechanics. 

In basketball, jumping is a commonly-performed maneuver during offensive and defensive plays. A basketball player performs an average of 44 jumps in a game and experiences impact up to nine-times their body weight during landing [10]. Repetitive jumps can impose excessive lower extremity joint loading that is not sufficiently attenuated by the soft tissues in a very short time interval [11], which thereby contribute to higher risks of ankle and impact related injuries [12,13]. Impact forces can be modulated by landing strategies [14,15], with larger ankle pronation leading to higher impact force and risk potential of non-contact anterior cruciate ligament (ACL) injury [16]. While neuromuscular training such as ankle disk training exercises would enhance joint coordination and proprioception at ankle joint [17], the use of foot insole was reported to induce acute changes in ankle [18], knee [19], and hip [20] mechanics that is related to lower risk of injuries [21]. However, it is still questionable if the sport insoles could minimize the injury risks in basketball. 

It is reported that more than 50% of basketball players use medial arch-support insoles in game play [22]. Biomechanically, arch-support insoles increase the foot-insole contact area and pressure at the medial longitudinal arch of a foot, which would enhance postural control and receptor sensory on the foot plantar during locomotion [16,23]. During drop landing, wearing foot insoles would reduce ankle eversion, knee valgus and loading, suggesting a lower risk of ACL injury during landing [24,25]. However, another study reported a higher maximum ankle inversion in arch-support insoles during landing [18]. The contradicting results could be due to the differences in landing types and intensities tested. Joseph et al. [24] used drop jump landing while Yu et al. [18] used a single-leg landing after a basketball lay-up in their studies. 

Another explanation could be related to the belief and expectation of the footwear tested [26]. Performance benefits of footwear under playing conditions can be maximized when one is confident with the purported benefits and performance-enhancing properties, as this was indicated by lighter shoe mass for higher jump height [27] and higher market price for better shoe comfort [28]. However, both Joseph et al. [24] and Yu et al. [18] studies did not measure subjective comfort variables. Subjective comfort has been received considerable attention by sports scientists and coaches. The foot insoles would increase subjective comfort and reduce pain/discomfort in various locomotion [22,29]. The increase of footwear comfort was related to higher performances [30] and lower incidence of injuries [31] in both training and competition in rugby. Studying both subjective comfort and movement mechanics could be necessary to determine the efficacy of foot insoles on jump landing performance in basketball. While wearing red is believed to be stronger and benefit performances (Sports [1]; Underwear-Mahjong superstitious [32]) and visual impacts due to footwear appearance would alter movement adaptation and control (e.g., visual pattern of shoe upper [33], shoe upper color [9]), there is a practical value in understanding the effect on comfort perception and landing biomechanics based on the red-insoles in basketball.

To date, while the majority of the investigations regarding foot insole effect on landing have been focused in basketball-playing females [19,20,24,25,34], little attention has been paid to the male population. It is reported that a greater number of participations and higher physical demands were found in males than females [35,36]. Furthermore, a basketball review indicated that only one out of seventeen studies investigated the effects of basketball footwear constructions in females, while the other studies investigated only in male population [37]. Therefore, studying foot insoles in males could benefit a wider population of basketball players. In addition, the previous studies reported that the differences in landing heights and/or landing types might have contributed to the distinct biomechanical and perceptual responses of different basketball footwear [38,39,40]. It should be noted that red color effect could be different among countries. For example, red-colored uniform was beneficial in Australian and English football [6], but no benefit was observed in German, Polish, and Spanish leagues [41,42]. Since previous studies reported that different perception and motor task performances were found in higher and lower gait speeds [33], studying arch-support insoles in different landing heights/intensities could help to understand the underlying mechanism of basketball landing in the Chinese population. 

Hence, the objective of this study was three-folded. The first objective of this study aimed to investigate whether arch-support insoles would influence impact forces, joint kinematics and kinetics, and comfort perception during landing from two landing heights. It is expected that arch-support insoles (white arch-support) would result in lower impact forces and joint loading while enhancing shoe comfort perception than the control insoles (white-flat). The second objective was to examine whether red color used (red arch-support) would have additional benefits over the white color insoles (white arch-support). Based on previous studies on colors and sport performances, red-colored insoles (red arch-support) are expected to have superior landing biomechanics and comfort perception than white color orthoses (white arch-support and white flat). The third objective was to examine if there was any interaction on test variables between foot insole and landing height. Landing from higher heights (i.e., impact intensities) may lead to distinct responses in subjective comfort and landing biomechanics than the lower heights, as indicated in the previous study [39]. The findings from this study can promote understanding when and how sports insoles should be used in basketball. 

## 2. Materials and Methods 

### 2.1. Participants

A priori power analysis was performed using G*power software (Dusseldorf, Germany) to determine the sample size. This was calculated from the previous studies with methods that closely related to this study [18,20]. Based on an alpha of 0.05 and 80% power, at least 15 participants were required. Therefore, nineteen male college basketball players from local sport universities [mean (SD) age = 22.0 (4.0) years, height = 1.80 (0.03) m, mass = 75.1 (7.6) kg] were recruited for this study. All of the participants were actively participating in collegiate basketball competitions for at least five years and not suffered from any lower extremity injury in the past six months. The inclusion criteria were foot length of US size 9, normal foot arch, and normal color vision. The foot length, foot arch, and color vision were confirmed with the Brannock foot measurement (Brannock Device, Syracuse, NY, USA), arch index assessment [43], and Ishihara color blindness tests [44], respectively. The participant was excluded if he has previous wearing experience of foot orthotic therapy/intervention to prevent any potential bias. The participants gave their informed consent for inclusion before they participated in the study. The study was conducted in accordance with the Declaration of Helsinki and the protocol was approved by the Ethics Committee of Li Ning Sports Science Research Center (IRB2017-BM014).

### 2.2. Foot Insole Conditions

**Figure 1:**
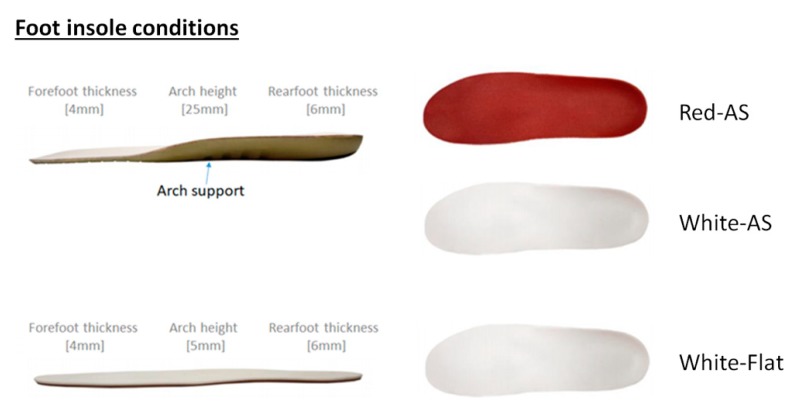
The thickness across forefoot, midfoot and rearfoot regions in each of three insole conditions (red arch-support (Red–AS), white arch-support (White–AS), white control (White–flat).

Three pairs of foot insoles were tested in this study (Figure 1): Red insole with arch-support (Red–AS), white insole with arch-support (White–AS), and white insole without arch-support (White–Flat). Both Red–AS and White–AS orthoses (Arch-support series-Universal II, Dr. Kong Footwear Ltd., Hong Kong, China) were made of polyurethane (PU) materials to allow uniform distribution of the foot plantar pressure and good shock absorption ability. Both Red–AS and White–AS orthoses were identical, except for the color of the top cover (Red vs. White). For the control condition (White–Flat), the flat insole used the same PU material and hardness as Red–AS and White–AS insoles while it did not have medial arch support. The material thickness across forefoot, midfoot and rearfoot regions are provided in Figure 1. 

### 2.3. Test Procedure

**Figure 2:**
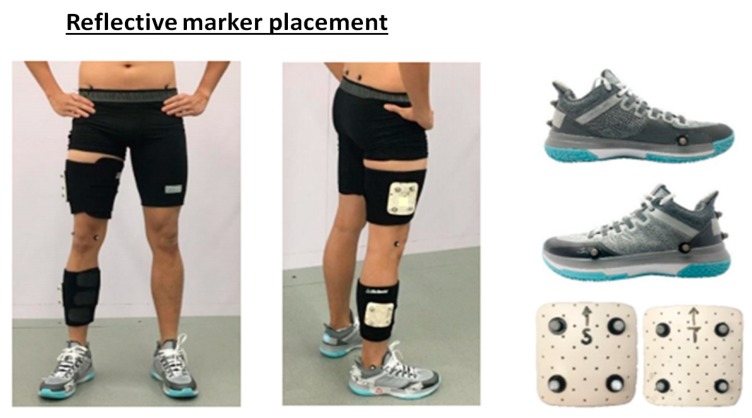
Reflective marker placements.

On arrival, anthropometric measurements of body mass, height, leg length, knee-width, and ankle-width were taken. Then, the participants performed 10-min warm-up and familiarized for drop landing tasks. The drop landing is a standard test to assess the cushioning capability of a shoe [38,40,45,46]. Reflective markers (diameter 14 mm) were placed over the following anatomical landmarks (Figure 2): Anterior and posterior superior iliac spines, medial and lateral epicondyles of femur, medial and lateral malleolus, three calcaneus markers (upper, lower, and lateral aspects of calcaneus), medial side of first metatarsal head, upper side of second metatarsal head, and lateral side of fifth metatarsal head, and two four-marker rigid clusters attached onto thigh and leg segments. The markers on the medial and lateral malleolus and femoral epicondyles were used during the static trial and then removed during landing.

The participants were asked to tighten their laces based on their individual preferences for basketball games. Prior to the actual testing, participants were reminded of the insole types in each orthosis condition, as it would encourage participants to be maximally aware of the functional benefits of the foot orthoses (color and contour) that led to the actual changes in performance [27,28,47]. The participants were asked to perform drop landing when wearing each of the three insoles (Red–AS, White–AS, and White-control) that were inserted into the same basketball shoe (Wade 4.0, Li Ning, Beijing, China). For the drop landing movement, participants were asked to stand straight and look forward while positioning their hands on the hips to minimize the influence due to arm movements. The movement was initiated when the participants were standing on a raised platform (0.45 and 0.61 m) above the ground and landed with their right leg on the force platform (AMTI, Watertown, NY, USA) and their left leg on the adjacent ground simultaneously [38,45]. The selected landing heights are commonly used in landing studies to compare differences in footwear conditions [38,39,45]. The synchronized force plate (AMTI, Watertown, NY, USA, sampling frequency of 1000 Hz) and 8-camera motion analysis system (Oxford Metrics Ltd., Oxford, UK, sampling frequency of 200 Hz) were used to collect the ground reaction force (GRF) and kinematic information during landing. 

Five successful trials were obtained for each of insole and landing height conditions. The successful trial was considered as correct foot placement contact with the center of the force plate and maintaining body balance during landing. The trial was discarded if an obvious loss of balance or discontinuity of movement was present. A 5-min rest period was given after each insole condition. During the rest period, participants were asked to rate their perceptions of forefoot cushioning, rearfoot cushioning, stability and overall comfort on a 15 cm visual analogue scale (VAS). The left end (0 cm) was labelled as “not very comfortable” and the right end (15 cm) as “very comfortable” [48]. The insole conditions were randomly assigned to participants. The comfort variables are the primary outcomes to assess the efficacy of foot insoles [47] and comfort level is related to the changes in sports performances [30].

### 2.4. Data Processing

The researcher (Y.W.) who was blinded to insole conditions performed data processing for this study. Marker trajectories were manually identified in Vicon Clinical Manager Software (Oxford Metrics Ltd., Oxford, UK) and a spline interpolation was performed using three frames before and after the missing data point. The marker trajectories were then smoothened using a fourth-order Butterworth low-pass digital filter. The cut-off frequency was determined using residual analysis, as described in previous studies [39] The contact period of the right leg was identified from the initial contact of one foot to 50 ms after maximum knee flexion [38,39,45]. The instance of foot contact was identified when the vertical GRF firstly exceeded 10 N (foot contact). A three-dimensional inverse dynamic model in Visual 3D (C-Motion Inc., ON, Canada), which comprised of the shoe, lower leg and thigh segments, was used for calculation of ankle and knee joint angles and moments. 

Ankle and knee joint angles were defined as the orientation of one distal segment (i.e., lower leg) relative to the proximal segment (i.e., thigh). A positive value for joint angle and moment denoted flexion, eversion, and internal rotation for respective orthogonal planes, with zero degree defined at a neural standing position for inversion–eversion and internal–external rotation. Joint range of motion was defined as the absolute difference between maximum flexion (inversion or internal rotation) and maximum extension (eversion or external rotation) [49,50]. All kinetic data was normalized to body weight. Ankle and knee joint biomechanics variables are direct relevance to the footwear assessment during jump and landing performance [38,45,51,52]. Peak vertical forefoot GRF, rearfoot GRF and maximum loading rates were also calculated, as these GRF variables are the key biomechanical indicators associated with landing injuries as well as foot insole effect [16,38]. 

### 2.5. Statistical Analysis

All statistical analyses were performed using SPSS 24 (IBM Corp., Armonk, NY, USA). Descriptive statistics (mean and standard deviation) of biomechanical and comfort perception variables were computed for respective insole and landing height conditions. The data normality was tested with Shapiro–Wilk tests. When Mauchley’s test indicated a violation of the sphericity assumption, Geisser’s epsilon adjustment was applied. For each test variable, separated 2 × 3 two-way (Insole x Landing height) ANOVA with repeated measures were employed to determine if there was any significant difference (*α* = 0.05), followed by Bonferroni post-hoc analyses for all tested variables. 

## 3. Results

### 3.1. Joint Angle Variables

No significant interaction between the insole and landing height (*p* > 0.05) or any main effect of orthosis (*p* > 0.05) were determined in ankle and knee kinematics variables (Table 1). At ankle joint, there were significant main effects on landing height for ankle eversion at touchdown, peak ankle eversion as well as sagittal ankle range of motion (RoM) *(p* < 0.01). Smaller eversion at touchdown and peak ankle eversion but larger sagittal ankle RoM were determined for higher landing height, as compared to lower landing height (*p* < 0.01). 

There were significant main effects of landing height for all knee variables (*p* < 0.01, Table 1). Post-hoc analyses revealed that participants landing from higher height demonstrated larger knee flexion at touch down, peak knee flexion and sagittal knee RoM, as compared to the landing from the lower landing height (*p* < 0.01). 

### 3.2. Ground Reaction Force Variables

We did not determine any significant interaction between the insole and landing height (*p* > 0.05) or any main effect of insole (*p* > 0.05, Table 2). Post-hoc analyses of landing height effect indicated higher forefoot GRF, rearfoot GRF and rearfoot maximum loading rate in higher landing height condition as compared to the lower landing height condition (*p* < 0.01).

### 3.3. Joint Moment Variables

**Figure 3:**
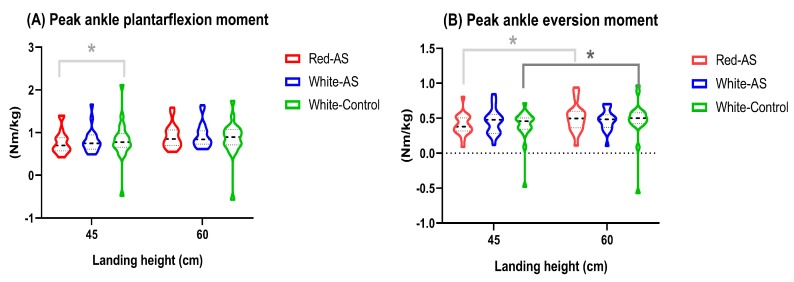
(**A**) Peak ankle plantarflexion moment and (**B**) peak ankle eversion moment by landing height and insole conditions.

There were significant interactions between insole and landing height for peak ankle plantarflexion and eversion moments (*p* < 0.05, Table 2 and Figure 3). The simple main effect revealed that participants wearing Red–AS insoles experienced smaller plantarflexion moment than White–Flat insoles (*p* < 0.01), but no differences were found between insoles when landing from higher landing height (*p* > 0.05). Additionally, wearing Red–AS and White–Control insoles were significant larger peak ankle eversion moment at higher than the lower landing height (*p* < 0.01, Figure 3), but no significant differences between landing heights were determined in White–AS insole (*p* > 0.05). 

The main effect of insole was determined for peak ankle plantarflexion moment (*p* = 0.03), such that participants wearing Red–AS insoles exhibited smaller plantarflexion moment than the White–Flat insoles (*p* < 0.05). In addition, higher peak ankle plantarflexion and eversion moments as well as knee extension moment were found at higher landing height as compared to the lower landing height (*p* < 0.01).

### 3.4. Subjective Comfort Variables

There were no significant interactions between insoles and landing height as well as the main effect of landing height for any perceptual variables (*p* > 0.05, Table 2). Main effects of insoles were determined for the perception of forefoot and rearfoot cushioning as well as overall comfort (*p* < 0.05). Post-hoc analyses revealed that participants wearing Red–AS insoles had better forefoot cushioning, rearfoot cushioning and overall comfort perceptions than the White–Flat insoles (*p* < 0.01). In addition, better forefoot cushioning perception was found in Red–AS insoles as compared to the White–Flat insoles (*p* < 0.01).

## 4. Discussion

Effects of color can arise from either seeing or wearing certain colors. Seeing color can act as a cue signal (similar to classical conditioning) while wearing colorful insoles can possibly affect the physical act of wearing. It helps the wearer to embody such qualities [1,4,5]. By Enclothed Cognition Theory, which proposes that the symbolic meaning of the clothes and the physical experience of wearing them can influence our cognition [53]. This study examined how different types of foot insoles (red arch-support, white arch-support, white-flat) tested would influence ground reaction forces, lower extremity kinematics and kinetics as well as comfort perception when landing from two landing heights. Our findings indicated that participants wearing Red–AS insoles experienced smaller plantarflexion moment than the ones wearing White–Flat insoles. Moreover, significant interactions between insole and landing height were found for peak ankle plantarflexion and eversion moments. Furthermore, participants perceived better comfort perception (i.e., forefoot cushioning, rearfoot cushioning, and overall comfort) in Red–AS than the White–Flat insoles.

Tibial stress fracture and knee injuries are the common overuse injuries in basketball. These injuries may relate to higher level of GRF and joint loading [13,21]. Previous studies have reported that arch-support insoles would provide mechanical support to the medial longitudinal arch of a foot and thus improve postural control and receptor sensory inputs onto the foot plantar in various functional tasks [16,23] and lower risk of knee injuries [21]. However, the reduction in GRF loading was not reported in arch-support insoles for healthy arched subjects in this study, which is partially aligned with the general understanding that with increased rigidity at midfoot/rearfoot by arch-support and/or ankle support, vertical impact loading increased at the early stance phase during running [54] and landing [55]. While external can effectively support the ligamentous structures in reducing overpronation during movements for a more neutral foot alignment [16,23], the kinetic energy originally absorbed by joint motion has to be released through increased impact force [55]. This is in contrast to other studies, which indicated that flatfooted individuals wearing arch-support insoles would experience lower vertical GRF and loading rates at heel contact in landing maneuvers [16]. The inconsistent GRF findings could be due to the differences in ankle motion characteristics between the normal and flatfooted athletes. This suggests that arch-support insoles could be more effective to reduce GRF loading in flatfooted individuals, rather than the normal arched individuals, in landing activities.

During drop landing, our participants wearing Red–AS insoles (i.e., Red arch-support) demonstrated smaller plantarflexion moment as compared to the White–Flat insoles (i.e., White–Flat), but there were no differences between White–AS and White–Flat insoles. These findings are in line with previous studies [24,25]. The reduced ankle eversion moment would be related to lower risk of ACL [52] and Achilles tendon injuries [56] in basketball. Moreover, lower peak ankle plantarflexion moment was found in Red–AS insoles than White–Flat insoles at lower landing height. Furthermore, participants perceived better forefoot cushioning, rearfoot cushioning, and overall comfort in Red–AS as compared to the White–Control insoles, although cushioning related parameters such as peak GRF and loading rates did not seem to be different. These significant differences only found in Red–AS, but not in White–AS, suggests that red color could enhance the positive effects when using arch-support insoles in basketball landing. 

Footwear comfort is considered as a prerequisite to minimize adverse effects on the human musculoskeletal system (impacts and stability) and enhance performance [30,46,57]. Increased comfort perception can lead to lower incidence of injuries [31], higher actual performance [30], minimized energy expenditures, and better footwear compliance [57]. Since the comfort level of footwear is considered to be a non-invasive and reliable measure of the potential risk of sports injuries, the relationship of psycho-motor effect requires further evaluation. One plausible explanation of Red–AS benefits is that psychological factors including the awareness and confidence in the benefits of footwear modification would maximize the influence of actual sports performances [26,27,28]. Another plausible mechanism is that visual/color perception would play some role in regulating the degree of comfort perception and movement control [33,58]. Considering the fact that all shoes tested had identical constructions (midsole thickness, hardness, and material properties), it suggests visual impacts due to insole colors may alter movement adaptation and control. Further studies should identify how the two psychomotor streams of colors can be applied in different sports movements. 

There are some limitations when interpreting our findings. First, only male basketball players with normal foot-arch were recruited. The findings may not be generalizable to female athletes or male athletes with lower foot arch, as biomechanical and perceptual data could be influenced by gender and foot morphology differences. It will be particularly important to acknowledge that red color effect was not consistent in football leagues across countries, such that wearing red-colored uniform was better in Australian and England leagues [6] while not superior in German, Polish, and Spanish leagues [41,42]. The color effect that exists is non-conclusive and are not related to different cultures. Second, although previous studies have reported that pre-fabricated arch-support insoles were equally as effective as custom-made foot insoles in producing changes in loading pattern during walking gait [59], it remains questionable whether using foot insoles custom made to individual foot shape may result in a larger degree of biomechanical differences that may in turn influence performance. Third, the participants were reminded about the foot insole conditions prior to the landing tasks. We expected that our participants would favor their positive expectation to the functional benefits of footwear and thus result in greater changes in actual performances, as described in previous studies [28,47]. Mohr et al. [27] reported that lightweight shoes leads to significantly better jump and shuffle cut performances than heavy shoes only when the participants were informed about the shoe mass differences (related to mechanical energy). No significant differences can be determined in the blinded group. Whether informed or blinded arch-support modification should be carried out before we could fully understand the psychomotor effect on the motor control. 

## 5. Conclusions

Higher landing heights were associated with higher joint loading and larger sagittal RoM, but smaller ankle eversion as compared to landing from lower landing heights. The application of colors in foot insole would play some roles in altering joint loading characteristics in basketball landing. Participants wearing red arch-support insoles induced smaller plantarflexion moment than those wearing white-flat insoles. Hence, altering color pattern can be implemented into insoles to influence the players’ comfort perception and joint loading, with some implications for risk of injury and footwear comfort in landing.

## Figures and Tables

**Table 1:** Ankle and knee kinematics of three insole conditions during the two landing heights.

Variable		Insole (I)			HxI	H	I
	Height (H)	Red–AS	White–AS	White–Flat	*p*	*p*	*p*
Ankle joint (°)							
Plantarflexion at touch down	Low	12.3 (7.6)	10.6 (10.4)	14.0 (8.8)	0.168	0.136	0.395
	High	12.4 (8.3)	13.9 (9.1)	14.1 (8.6)			
Eversion at touch down	Low	4.9 (4.1)	5.0 (5.5)	4.4 (4.5)	0.125	**<0.001**	0.803
	High	4.4 (4.0)	3.4 (5.1)	4.2 (4.8)			
Peak dorsiflexion	Low	28.6 (6.4)	29.2 (8.4)	28.0 (6.6)	0.299	0.168	0.842
	High	29.7 (5.8)	28.8 (8.3)	29.5 (7.4)			
Peak eversion	Low	3.6 (3.0)	2.6 (4.0)	3.5 (3.7)	0.754	**0.001**	0.204
	High	2.9 (3.1)	1.8 (3.9)	2.8 (3.9)			
RoM-sagittal	Low	39.2 (7.8)	38.5 (7.3)	38.9 (7.6)	0.517	**<0.001**	0.816
	High	42.4 (7.2)	42.5 (6.9)	43.4 (6.9)			
RoM-coronal	Low	8.6 (2.5)	9.2 (3.8)	8.3 (2.6)	0.166	0.135	0.827
	High	9.4 (2.7)	9.1 (2.3)	9.4 (2.6)			
Knee joint (°)							
Flexion at touch down	Low	20.4 (5.5)	20.5 (5.8)	21.2 (5.0)	0.648	**<0.001**	0.540
	High	23.7 (4.9)	23.6 (6.5)	25.0 (6.1)			
Peak flexion	Low	86.0 (13.9)	83.9 (17.7)	85.3 (15.3)	0.499	**<0.001**	0.567
	High	93.8 (14.5)	91.0 (18.1)	94.1 (15.6)			
RoM-sagittal	Low	83.8 (14.5)	81.1 (19.8)	82.5 (18.7)	0.472	**<0.001**	0.469
	High	92.5 (16.3)	87.9 (20.3)	90.6 (17.9)			

RoM = total range of excursion; HxI = interaction effect; H = height effect; I = insole effect. Significant differences (*p* < 0.05) are shown in bold.

**Table 2:** Ground reaction force (GRF), joint moment and comfort perception of three insole conditions during the two landing heights.

Variable		Insole (I)			HxI	H	I
	Height (H)	Red–AS	White–AS	White–Flat	*p*	*p*	*p*
GRF (BW)							
Forefoot peak vGRF	Low	1.11 (0.60)	1.10 (0.28)	1.06 (0.32)	0.859	**<0.001**	0.533
	High	1.42 (0.36)	1.37 (0.37)	1.32 (0.36)			
Rearfoot peak vGRF	Low	3.51 (0.60)	3.63 (0.75)	3.55 (0.75)	0.710	**<0.001**	0.642
	High	4.05 (0.63)	4.12 (0.82)	4.01 (0.73)			
Rearfoot max LR (BW/s)	Low	467.10 (190.11)	453.50 (195.70)	433.97 (177.36)	0.110	**<0.001**	0.37
	High	593.43 (231.34)	500.30 (171.31)	534.66 (230.58)			
Joint moment (Nm/kg)							
Peak plantarflexion moment	Low	0.77 (0.27)	0.81 (0.28)	0.89 (0.38)	**0.005**	**<0.001**	**0.034**
	High	0.91 (0.28)	0.93 (0.29)	0.93 (0.30)			
Peak ankle eversion moment	Low	0.40 (0.16)	0.46 (0.18)	0.44 (0.14)	**0.032**	**<0.001**	0.733
	High	0.51 (0.21)	0.47 (0.15)	0.52 (0.18)			
Peak knee extension moment	Low	2.06 (0.60)	2.13 (0.68)	2.06 (0.61)	0.152	**<0.001**	0.910
	High	2.66 (0.82)	2.50 (0.81)	2.63 (0.82)			
Comfort perception							
Forefoot comfort perception	Low	9.46 (2.58)	7.14 (2.17)	7.34 (2.62)	0.233	0.437	**<0.001**
	High	8.83 (2.51)	7.43 (2.42)	7.07 (2.72)			
Rearfoot comfort perception	Low	9.71 (2.56)	8.75 (2.53)	7.02 (1.97)	0.127	0.481	**<0.001**
	High	9.33 (2.83)	8.12 (2.77)	7.42 (3.04)			
Stability perception	Low	9.65 (2.38)	9.41 (2.46)	8.87 (2.56)	0.656	0.544	0.142
	High	9.70 (2.42)	9.05 (2.59)	8.72 (2.22)			
Overall comfort perception	Low	9.76 (2.18)	8.88 (2.69)	7.64 (2.14)	0.362	0.192	**0.005**
	High	9.53 (2.47)	8.16 (2.46)	7.68 (2.32)			

HxI = interaction effect; H = height effect; I = insole effect. Significant differences (*p* < 0.05) are shown in bold.
